# Collateral effects of the SARS-CoV-2 pandemic on oncologic surgery in Bavaria

**DOI:** 10.1186/s12893-021-01404-y

**Published:** 2021-12-04

**Authors:** Thomas Dienemann, Frank Brennfleck, Alexander Dejaco, Robert Grützmann, Johannes Binder, Christian Krautz, Christian Stöß, Carsten Jäger, Helmut Friess, Hans Jürgen Schlitt, Stefan M. Brunner

**Affiliations:** 1 Klinik und Poliklinik für Chirurgie, Universitätsklinikum Regensburg, Franz-Josef-Strauß-Allee 11, 93053 Regensburg, Germany; 2 Klinik für Anästhesiologie, Universitätsklinikum Regensburg, Regensburg, Germany; 3 Chirurgische Klinik, Universitätsklinikum Erlangen, Friedrich-Alexander-Universität Erlangen-Nürnberg, Erlangen, Germany; 4 Department of Surgery, Klinikum rechts der Isar, School of Medicine, Technical University of Munich, Munich, Germany

**Keywords:** SARS-CoV-2, COVID-19, Oncologic surgery, Oncology, Patient care

## Abstract

**Background:**

The ongoing SARS-COV-2 pandemic has severe implications for people and healthcare systems everywhere. In Germany, worry about the consequences of the pandemic led to the deferral of non-emergency surgeries. Tumor surgery accounts for a large volume in the field of visceral surgery and cannot be considered purely elective. It is not known how the SARS-COV-2 pandemic has changed the surgical volume in tumor patients.

**Methods:**

Retrospective analysis of the amount of oncological surgeries in three academic visceral surgery departments in Bavaria, Germany, in 2020. Procedures were split into subgroups: Upper Gastrointestinal (Upper GI), Colorectal, Hepato-Pancreato-Biliary (HPB), Peritoneal and Endocrine. Procedures in 2020 were compared to a reference period from January 1st, 2017 to December 31st 2019. Surgical volume was graphically merged with SARS-COV-2 incidence and the number of occupied ICU beds.

**Results:**

Surgical volume decreased by 7.6% from an average of 924 oncologic surgeries from 2017 to 2019 to 854 in 2020. The decline was temporally associated with the incidence of infections and ICU capacity. Surgical volume did not uniformly increase to pre-pandemic levels in the months following the first pandemic wave with lower SARS-COV-2 incidence and varied according to local incidence levels. The decline was most pronounced in colorectal surgery where procedures declined on average by 26% following the beginning of the pandemic situation.

**Conclusion:**

The comparison with pre-pandemic years showed a decline in oncologic surgeries in 2020, which could have an impact on lost life years in non-COVID-19 patients. This decline was very different in subgroups which could not be solely explained by the pandemic.

## Background

The ongoing SARS-CoV-2 pandemic has severe implications for people and healthcare systems all over the world. Estimates show that years of life lost exceed 300,000 years in Germany alone in 2020. Every deceased person lost on average 9.6 years of life [1]. However, this pandemic also affected patients with other diseases who have not been infected with the virus.

The German Government implemented a lockdown on March 16 of 2020 due to concern of exceeding the threshold capacity of the healthcare system. Simultaneously, due to an increased demand and a reduction of commerce there were shortages of personal protective equipment, single use items such as ventilator filters and medication. Additionally, the German Government prompted hospitals to reserve beds to be able to respond to a possible surge in COVID-19 hospital admissions. This led to the deferral of non-emergency surgical procedures. The consequences on clinical outcomes in patients with oncological diseases have yet to be elucidated.

In Germany, a first wave of infections peaked on April 1st 2020 with 1991 infections in Bavaria in a single day and inpatient COVID-19 cases peaked roughly 2 weeks later. A second wave slowly emerged during the late summer and reached its peak in late December of 2020.

Multiple groups reported survey data of reduced surgical volume during spring and summer of 2020 [2–4] in Germany and around the world [5, 6]. Additionally, the overall observed patient volume also declined during the peak of the pandemic in spring in Germany [7, 8]. This was not only observed in fields where the majority of patients are treated electively but also in fields with a high volume of acute care such as cardiology and neurology [9, 10].

SARS-COV-2 infection rates were unevenly distributed throughout Germany and Bavaria during the first wave, and hospital and ICU occupancy also varied widely locally. 

In the present study, we examine the relationship between the oncologic surgery volume in three academic visceral surgery departments and state-wide SARS-CoV-2 incidence rates in the state of Bavaria in Germany.

## Methods

In this retrospective study, data were collected from hospital discharge data of three Bavarian university hospitals (Klinikum rechts der Isar der Technischen Universität München (MRI TUM), Universitätsklinikum Erlangen (UKER), and Universitätsklinikum Regensburg (UKR)) between January 1, 2017 and December 31, 2020. The dataset contained structured data on the exact diagnosis, type and date of the procedure from the International Classification of Diseases (ICD) and the German coding system for operations and procedures (OPS). Procedures from January 1st, 2017 to December 31st, 2019 were averaged and served as a historical comparator (reference period) against the entire year of 2020, the pandemic period. This study only included patients with an oncologic diagnosis. Procedures were divided into the following groups: Upper Gastrointestinal (Upper GI), Colorectal, Hepato-Pancreato-Biliary (HPB), Peritoneal and Endocrine to compare changes in frequencies in subgroups between years. This dataset was combined with daily Bavarian SARS-CoV-2 infection rates according to data from the Bavarian State Office for Health and Food Safety for graphical analysis [11] and with the number of vacant ICU beds, which were publicly accessible through the German Interdisciplinary Association for Intensive Care and Emergency Medicine (DIVI) [12]. This service was made available in late March of 2020. In order to be able to statistically compare count data in surgical procedures between the reference period and the year 2020, the reference period and the pandemic year were split into 4 periods (January-March, April-June, July–September, and October-December). The quarters April to June of 2020 and October to December of 2020 correspond to the first and (the beginning of) the second wave in Germany. We regarded the number of surgeries in each quarter as counts that arise from a Poisson process. The person time needed in order to obtain incidence rate ratios was computed from the population at risk (i.e. the total population in Bavaria) for each quarter. The total population for each quarter is provided by the “Bevölkerungsstatistischer Quartalsbericht” from the Bavarian State Office for Statistics (Bayerisches Landesamt für Statistik) [13].

Descriptive data are presented as count data. A Chi-squared test was used to for hypothesis testing, a p-value < 0.05 was considered statistically significant and all tests were two tailed. Stata 14 (Stata Corp., USA) was used for statistical analyses and. MATLAB (The Mathworks Inc., USA) was used for all graphical analyses.

No informed consent or ethical approval was required because fully anonymized data was used. The collection and use of anonymized data for research purposes are regulated under the Bavarian Hospital Law (Bayerisches Krankhausgesetz Art. 27 Abs. [4]).

## Results

The differences in surgeries per center over the past 4 years can be seen in Table 1. The volume of oncologic surgery cases in 2020 decreased in two centers (UKR − 14%, UKER − 12%), whereas it slightly increased in one center (MRI TUM + 5%).

**Table 1:** All surgeries, by center, % change gives the change in procedures from the 2017–2019 period compared to the 2020 period

Center	2017	2018	2019	Avg. years 2017–2019	2020	% Change
UKR	304	307	248	286	244	− 14%
UKER	347	357	390	366	321	− 12%
TUM MRI	272	276	275	274	289	+ 5%
Total	923	935	913	923.6	854	− 7%

The average number of surgeries per year from 2017 to 2019 across all three centers was 923.6. In 2020, the three centers reached a total number of surgeries of 854. This marks a decrease of approximately 7%. Table 2 presents differences in procedures by group from the average of all three centers from 2017 to 2019 (reference period) in comparison to 2020. Upper GI procedures were similar in numbers in the first quarter of 2020 compared to the reference period but severely dropped in the second quarter (− 31%). Procedures increased in the second quarter (+ 7%) but decreased again in the third quarter (− 3.7%). None of these changes reached a statistically significant difference. Colorectal procedures in 2020 did not differ in comparison to the reference period. The number of procedures dropped in the second quarter by 13%, in the third quarter by 31% and by 34% in the third quarter. Whereas the change in the second quarter was not statistically different, the reduction in the third and fourth quarter were significant. HPB procedures increased in the first quarter of 2020 in comparison to the reference period. HBP surgery dropped numerically in the second quarter but increased again in the third quarter only to decrease again in the last quarter of 2020. Despite the nomically large differences none of the changes was statistically different. Due to the low volume of endocrine and peritoneal surgeries, the relative differences are inflated in these subgroups.

**Table 2:** Changes per quarter (average of 2017–2019) in relation to 2020**,** RRD: relative rate difference (≙ incidence rate ratio) in percent

Type of surgery	17–19/1	2020/1	RRD% (p-value)	17–19/2	2020/2	RRD% (p-value)	17–19/3	2020/3	RRD% (p-value)	17–19/4	2020/4	RRD% (p-value)
Upper GI	47	46	− 3% (0.445)	45	31	− 31% (0.050)	48	52	7 (0.360)	40	39	− 3.7% (0.441)
Colorectal	98	99	0.4% (0.490)	83	73	− 13% (0.200)	104	73	− 31% (0.008)	87	58	− 34% (0.007)
HPB	75	95	+ 30% (0.068)	82	63	− 24% (0.053)	78	92	15 (0.154)	78	65	− 18% (0.129)
Peritoneal	4	3	− 25% (0.360)	4	2	− 50% (0.224)	4	4	± 0 (0.495)	4	6	32% (0.278)
Endocrine	8	12	33% (0.195)	10	8	− 25% (0.318)	11	6	− 44% (0.115)	12	0	–
All surgeries	232	256	+ 9% (0.156)	221	177	− 20% (0.011)	247	235	− 6% (0.263)	218	169	− 23% (0.005)

Means were rounded to the nearest whole number

There were large differences among hospitals in yearly procedures broken down into subgroups even in the years before 2020. For example, the absolute number of HPB procedures at UKR varied between 127 and 161 per year, representing a relative difference of 21%. An even greater difference was seen in the colorectal subset, where operations decreased by 26% from 2018 to 2019. Similar differences were observed at MRI TUM and UKER. At MRI TUM, yearly colorectal procedures showed a variability with a relative difference from 2017 to 2019 of 17%. At UKER, colorectal procedures increased by 18% from 2017 to 2019 (absolute 174 to 213). In the pandemic year 2020, colorectal procedures dropped by 23% compared to 2019.

HPB procedures remained somewhat more stable with smaller relative differences between the years. Upper-GI also remained relatively stable at TUM and UKER, whereas at UKR procedures decreased by 46% between years 2017 and 2019. Center-level subgroups can be viewed in Table 3.

**Table 3:** All centers, total surgeries years 2017–2020

Center	Type of surgery	2017	2018	2019	2020
UKR	Upper GI	41	35	22	30
	Colorectal	94	95	70	76
	HPB	140	161	127	122
	Peritoneal	7	4	4	5
	Endocrine	22	12	25	11
	Total	304	307	248	244
TUM MRI	Upper GI	88	93	94	81
	Colorectal	97	90	80	78
	HPB	67	76	83	111
	Peritoneal	13	9	7	9
	Endocrine	14	8	11	10
	Total	272	276	275	289
UKER	Upper GI	59	45	63	62
	Colorectal	174	209	213	162
	HPB	101	92	102	87
	Peritoneal	3	3	7	5
	Endocrine	10	3	5	5
	Total	374	357	390	321

**Fig. 1:**
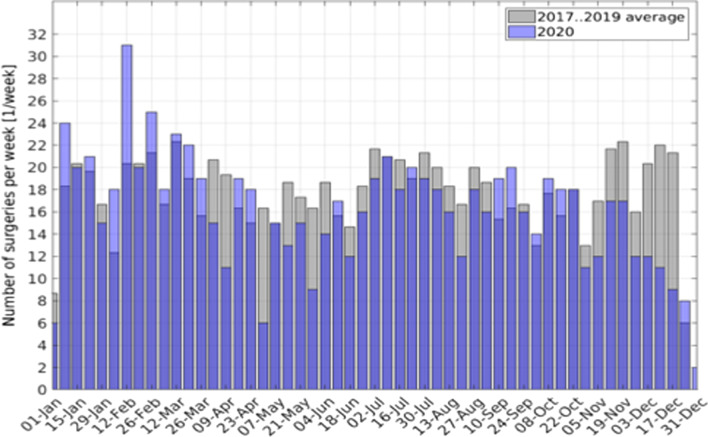
Weekly surgical volume of the average of the years 2017 to 2019 compared to weekly surgical volume in 2020

**Fig. 2:**
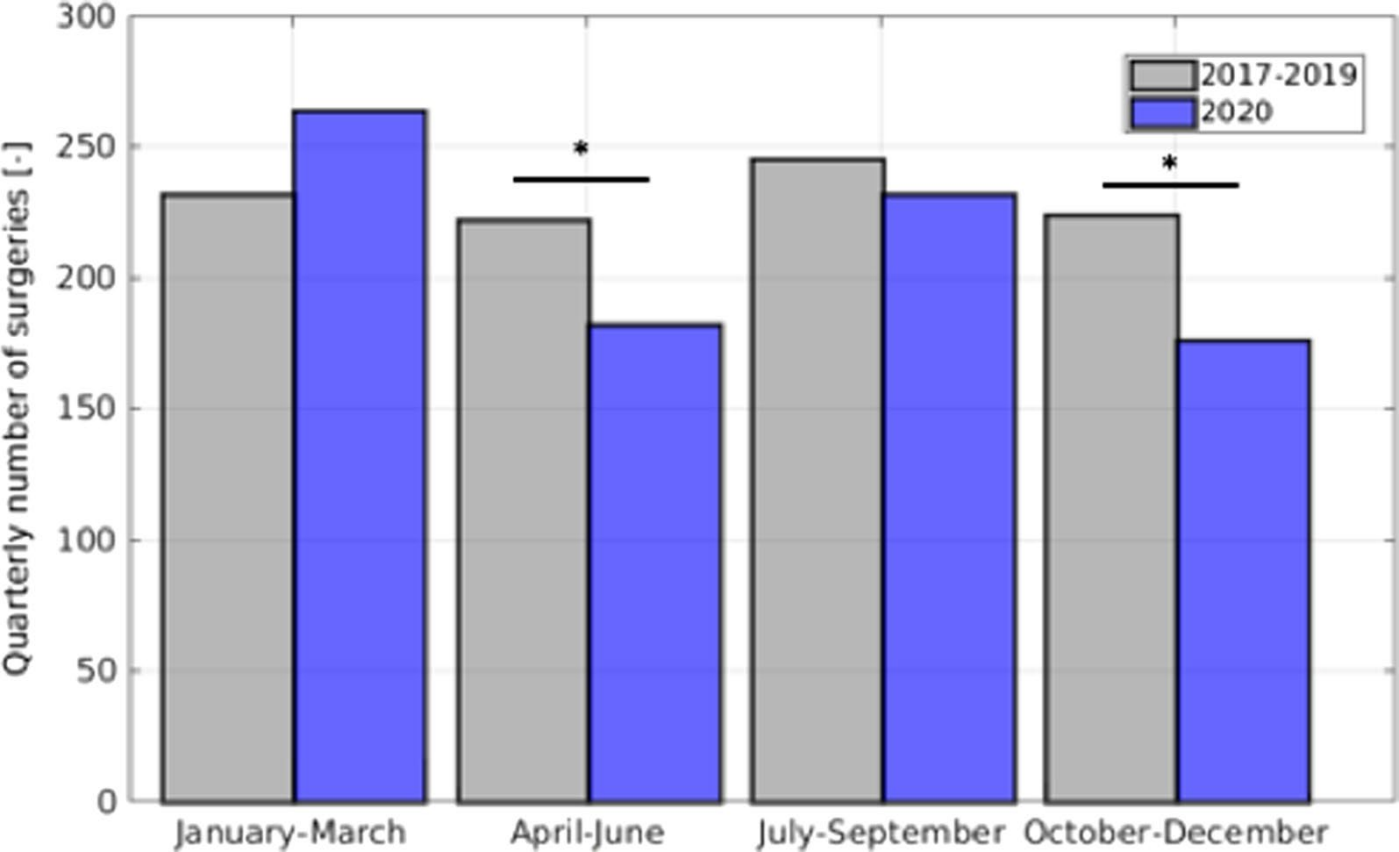
Quarterly surgical volume of the average of the years 2017 to 2019 compared to quarterly surgical volume in 2020, Asterisk marks a statistically significant difference

Figure 1 shows the weekly number of surgeries in the years 2017 to 2019 compared to weekly surgeries in 2020. In 2020, surgical volume appeared to be much more unsteady compared to 2017–2019. A marked decline became apparent around calendar week 16, with surgical volume remaining lower for several weeks until calendar week 24. From then on, surgical volume did not reach the levels observed in the first 12 weeks of 2020 or compared to similar time points in the aggregate data from 2017 to 2019. Surgical volume seemed to be steadily decreasing in the last quarter of 2020, which is in contrast to a relatively stable number in previous years. The surgical volume by quarter is depicted in Fig. 2. The average in surgical volume of 2017–2019 appears to be relatively consistent on a quarterly basis. The average spans from 218 to 247 surgeries per quarter. In 2020 the quarterly surgeries are as many as 256 in the first quarter and as few as 169 in the fourth quarter.

The differences in surgical volume from 2017 to 2019 compared to 2020 is significant in the second and in the third quarter.

**Fig. 3:**
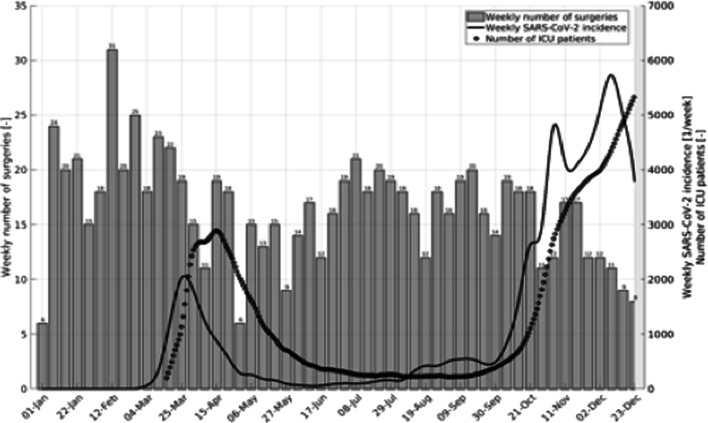
Weekly incidence of SARS-CoV-2 cases, ICU beds* occupied by SARS-COV-2 patients and surgeries per week in all three centers in 2020. *Service was made available in April of 2020

Figure 3 shows weekly SARS-CoV-2 cases and occupied ICU beds by patients with COVID-19 compared to weekly surgical volume from all three centers in Bavaria in 2020. The drop in surgical volume tends to trail incidence rates with a time lag of 2 to 3 weeks. In the second half of 2020, surgical volume seemed to drop slowly, contrasting with the increasing incidence and ICU occupancy.

## Discussion

The present study evaluated the association between the incidence rates of SARS-CoV-2 and oncological surgeries in three visceral surgery departments in academic centers in the state of Bavaria, Germany, which was severely affected by a high SARS-CoV-2 incidence in 2020.

Despite annual fluctuations in surgical volume in the years preceding the pandemic, there was a significant overall decrease in oncologic surgical procedures in 2020.

Weekly surgical procedures were at similar levels compared to previous years during the first three months of 2020, but then declined sharply in April and May 2020 in reciprocity with SARS-CoV-2 incidence. After this first wave, surgical volume increased but did not reach the levels of previous years. A second continuing decline in surgical volume began in late October 2020, as incidences of SARS-CoV-2 started to rise again.

There were large differences when assessing the number of procedures in the subgroups. The number of procedures declined in all groups during the first wave. Multiple factors lead to this: First, at the beginning of this pandemic, there were fears of a shortage of personal protective gear and other disposable equipment, as well as fears of an overburdened health system. In an effort to minimize the use of disposable equipment, the government and surgical societies called for the suspension of elective and nonessential surgeries. Second, to handle a potential rising number of patients, operating rooms were closed, and staff were reassigned to help in ICUs and exclusive COVID-19 floors. In some hospitals, the post-anesthesia care unit was also converted to an ICU, further limiting the amount of possible surgical procedures. Third, there was also a measurable reluctance to seek medical care on the patient side, most likely due to fear of acquiring an infection during hospitalization. Awareness of an ongoing pandemic probably increased drastically as the German Government declared the first nationwide lockdown from March 16 to May 4, 2020.

At the end of the first lockdown, the number of daily surgeries increased. This was probably due to ensured supply chains of disposables, a sharp decrease in incidence and a decline, albeit delayed, in COVID-19 patients in ICUs. Interestingly, the number of daily surgeries did not reach pre-pandemic levels. The analysis in the subgroups showed a recovery in upper GI and HPB surgery during the third quarter and second albeit smaller decrease in the fourth quarter. The amount of colorectal procedures decreased less than HPB and upper GI surgery during the first wave but contrary to the other two remained low in the third quarter and showed the highest decline of all subgroups during the second wave which was the main reason for the continued decrease in overall surgeries.

A general decrease in procedures is in line with many previous reports which show fewer hospital admissions in many elective fields [7]. A study by Richter et al. showed a decline in hospital admissions for strokes, transient ischemic attacks and intracerebral hemorrhages of − 18%, − 22.9% and − 15.8%, respectively, during the weeks of the first lockdown in Germany [9]. A similar report was published by De Rosa et al. who reported a 48.5% decline of hospitalizations for myocardial infarction in Italy in March 2020 [10]. These numbers are especially alarming since a reduction of elective treatments due to the imposed policies was expected but strokes and myocardial infarction are diseases that due to their physiology should not be significantly affected by lockdown measures.

The persistent decrease in colorectal surgery could have been due to a change in treatment strategies because of the pandemic and its consequences such as continued decrease in surgical capacity. A definitive explanation cannot be derived from the present data. The increase in HBP surgery was probably unrelated to the SARS-CoV-2 pandemic. A new law came into effect in 2020 which demanded a yearly minimum of complex procedures especially in HBP surgery in order to be eligible for full re-imbursement by insurers. This could have led to a decline of these procedures in smaller hospitals and a subsequent increase in high care centers. The differences in overall surgical volume in the three participating hospitals are most likely multifactorial. The pre-pandemic differences can be explained by differences in surgical spectrum, differences in department and hospital size and natural annual fluctuations.

In regard to the pandemic period, several additional factors have to be considered when comparing surgical volume in these centers. A major factor was the strategy which hospitals used to cope with the surge in COVID-19 patients. The participating hospitals were individually responsible for adequate planning and the distribution of COVID-19 patients within a hospital was different in all three participating centers.

There were furthermore great differences in local incidence rates, especially during the first wave. Hotspots with high incidences per capita were in regions bordering the Czech Republic, which are quite close to UKR.

In addition, the geographic regions of the participating hospitals vary greatly. MRI TUM is located in Munich, the largest and most populous city in Bavaria with approximately 2.9 million residents in the city and greater Munich area [13]. However, Munich has a second university hospital and four other tertiary care hospitals, so patients were likely more evenly distributed. This may have been an additional reason for the increase in operations at TUM during 2020. UKER is located in a much less populated area in Bavaria with 2 large hospitals within a 30 km radius and around 1.3 million inhabitants in the greater area [13]. UKR is located, similar to UKER, in a less densely populated region, but is the only tertiary care center in a region (Upper Palatinate, Oberpfalz) with about 1.8 million residents [13]. Due to the fact that UKR is a supraregional center for ECMO therapy, the admission radius is around 250 km.

The effect of the pandemic precautions on oncologic surgical patients cannot yet be measured. It is not known whether postponing urgent oncologic treatment resulted in worse outcomes. It is also unclear whether oncologic patients who would have been candidates for surgery where referred to oncology due to reduced operating room and ICU capacities. However, the total difference of 69.6 surgeries from the 2017–2019 mean to the 2020 mean corresponds to a regular operating volume of almost four working weeks in all three centers. Within four weeks, it is possible that tumors progress significantly, which may lead to irresectable operative situations and ultimately result in additional cancer deaths and life-years lost. It has been shown that in the United Kingdom, an average presentation delay of 2 months per patient can result in 181–542 additional deaths and 3316–9948 life-years lost, depending on the extent of the case backlog [14]. These results are confirmed by another study using data of 24,975 colorectal and 6744 esophageal cancer patients which calculated an increase in deaths of 15.3–16.6% for colorectal and 5.8–6.0% for esophageal cancers up to 5 years after the diagnosis. For these two tumors types, these data correspond to 1775 additional deaths [15]. To address these delays and prevent some of the additional deaths, an additional capacity over a 3-months period would be necessary [14]. However, this was not the case in the 3 academic centers studied in Bavaria, and therefore, a relevant increase of cancer deaths and lost life years as a result of the lockdown situation could be expected. A possible solution to better direct patients prior to planned surgery in a pandemic by telephonic triage was proposed by an Italian group early in the pandemic [16]. The same group was also among the earliest to publish general recommendations to reduce contagions in hospitals and decrease risk of infections among patients [17].

This study has several limitations. From the data presented it cannot be ruled out that causes unrelated to the pandemic and not mentioned in the discussion may also have played a role in the reduction of surgical volume in 2020. The selection of these three centers was not random, so generalizability cannot be warranted. Data on surgeries were available only by ICD 10 and OPS codes. Individual patient data such as age, sex and disease stage were not available to analyze differences in cohorts in further detail.

## Conclusion

The present study demonstrates a reduction in the treatment of visceral oncologic surgical patients in 2020, and the ramifications of this pandemic will likely be felt for the foreseeable future and will undoubtedly have a momentous effect on health care. The years of life lost by non-COVID-19 patients is a factor that has to be considered in the future management of pandemics and, as short-term measure to limit these losses, may be prevented by better directing patients and increasing capacities for cancer patients immediately after lockdown periods.

## Data Availability

The datasets used and/or analyzed during the current study are available from the corresponding author on reasonable request.

## Abbreviations

DIVI: German Interdisciplinary Association for Intensive Care and Emergency Medicine
HPB: Hepato-pancreato-biliary
ICU: Intensive care unit
ICD: International Classification of Diseases
MRI-TUM: Klinikum rechts der Isar der Technischen Universität München
OPS: German coding system for operations and procedures
RRD: Relative rate difference (≙ indicence rate ratio)
SARS-CoV-2: Severe acute respiratory syndrome-corona virus-2
Upper GI: Upper gastrointestinal
UKER: Universitätsklinikum Erlangen
UKR: Universitätsklinikum Regensburg
